# Assessing the genetic diversity of farmed and wild Rufiji tilapia (*Oreochromis urolepis urolepis*) populations using ddRAD sequencing

**DOI:** 10.1002/ece3.6664

**Published:** 2020-08-18

**Authors:** Christer S. Nyinondi, Matern S. P. Mtolera, Aviti J. Mmochi, Fernando A. Lopes Pinto, Ross D. Houston, Dirk J. de Koning, Christos Palaiokostas

**Affiliations:** ^1^ Department of Animal Breeding and Genetics Swedish University of Agricultural Sciences Uppsala Sweden; ^2^ Institute of Marine Sciences University of Dar es Salaam Zanzibar Tanzania; ^3^ The Roslin Institute and Royal (Dick) School of Veterinary Studies University of Edinburgh Edinburgh UK

**Keywords:** ddRAD‐seq, genetic diversity, Rufiji tilapia

## Abstract

Rufiji tilapia (*Oreochromis urolepis urolepis*) is an endemic cichlid in Tanzania. In addition to its importance for biodiversity conservation, Rufiji tilapia is also attractive for farming due to its high growth rate, salinity tolerance, and the production of all‐male hybrids when crossed with Nile tilapia (*Oreochromis niloticus*). The aim of the current study was to assess the genetic diversity and population structure of both wild and farmed Rufiji tilapia populations in order to inform conservation and aquaculture practices. Double‐digest restriction‐site‐associated DNA (ddRAD) libraries were constructed from 195 animals originating from eight wild (Nyamisati, Utete, Mansi, Mindu, Wami, Ruaha, Kibasira, and Kilola) and two farmed (Bwawani and Chemchem) populations. The identified single nucleotide polymorphisms (SNPs; *n* = 2,182) were used to investigate the genetic variation within and among the studied populations. Genetic distance estimates (*F*_st_) were low among populations from neighboring locations, with the exception of Utete and Chemchem populations (*F*_st_ = 0.34). Isolation‐by‐distance (IBD) analysis among the wild populations did not detect any significant correlation signal (*r* = .05; *p*‐value = .4) between the genetic distance and the sampling (Euclidean distance) locations. Population structure and putative ancestry were further investigated using both Bayesian (Structure) and multivariate approaches (discriminant analysis of principal components). Both analysis indicated the existence of three distinct genetic clusters. Two cross‐validation scenarios were conducted in order to test the efficiency of the SNP dataset for discriminating between farmed and wild animals or predicting the population of origin. Approximately 95% of the test dataset was correctly classified in the first scenario, while in the case of predicting for the population of origin 68% of the test dataset was correctly classified. Overall, our results provide novel insights regarding the population structure of Rufiji tilapia and a new database of informative SNP markers for both conservation management and aquaculture activities.

## Graphical abstract

The objective of the current study was to assess the genetic variation among 10 Rufiji tilapia populations of both wild and farmed origin using ddRAD‐seq. We investigate for the existence of putative genetic clusters, test for the existence of isolation by distance, and assess the efficiency of predicting population of origin‐based only on the genomic profile using cross‐validation schemes.

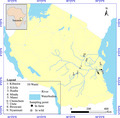

## 1 INTRODUCTION

Tilapias (Cichlidae family) comprise a diverse group of over 70 species mostly encountered in tropical and subtropical regions (McAndrew & Majumdar, 1983; Trewavas, 1983). Native in a diverse range of habitats across Africa, they are particularly important in the biodiversity of freshwater ecosystems. Moreover, tilapias are of paramount value for the aquaculture industry, being cultured in over 120 countries with a global production volume exceeding 5 million tonnes (FAO, 2018). Overall, tilapia aquaculture production is dominated by Nile tilapia (*Oreochromis niloticus*) farming which has been introduced in a wide range of habitats worldwide. Nevertheless, the impact to the local fauna is in many cases poorly understood (Lima, Oliveira, Giacomini, & Lima‐Junior, 2018) even though concerns have been raised (Canonico, Arthington, Mccrary, & Thieme, 2005). Furthermore, prior experience from several aquatic species suggests that introduced species can negatively affect biodiversity (Lovell, Stone, & Fernandez, 2006).

Tanzania is a hot spot for tilapias, with current knowledge suggesting that 10 Oreochromis species are endemic only to the country (Genner, Turner, & Ngatunga, 2018). In an attempt to boost the productivity of local fisheries and aquaculture farms, *Oreochromis* species like the Nile tilapia (endemic only to Lake Tanganyika) have been introduced to non‐native habitats across the country often in an unregulated manner (Kajungiro, Mapenzi, et al., 2019). Recent studies posed concerns regarding the negative impact toward the local fish fauna due to the introduction of Nile tilapia to non‐native habitats (Gu et al., 2017; Padial et al., 2017; Rico‐Sánchez et al., 2020).

Furthermore, interspecific hybridization is common among *Oreochromis* species (Scribner, Page, & Bartron, 2000) with fertile hybrids occurring either spontaneously in the wild or due to aquaculture practices that aim to improve desirable traits in farmed stocks like growth and salinity tolerance (Kamal & Mair, 2005). Therefore, hybridization between introduced and native tilapia species can severely impact the unique genetic diversity of the latter affecting their adaptation capacity toward changing environmental conditions (Deines, Wittmann, Deines, & Lodge, 2016). Even though the exact consequences of introduced tilapia* *species in Tanzania to the local fauna are unknown, habitat loss and significant decline of population size have been recently documented for the endemic *Oreochromis hunter *in Lake Chala, in Kilimanjaro Tanzania due to introduced tilapia species (Moser, van Rijssel, Ngatunga, Mwaiko, & Seehausen, 2019).

Rufiji tilapia (*O. urolepis urolepis*) is an endemic species in Tanzania, distributed mainly across the south‐eastern rivers, reservoirs, and oxbow lakes of Rufiji river basin (Ulotu, Mmochi, & Lamtane, 2016). Interestingly, according to Genner et al. (2018) the Wami, Zanzibar, and Rufiji tilapia all refer to the same species. Over the years, nonendemic species like the Nile tilapia and the blue‐spotted tilapia (*Oreochromis leucostictus*) have been introduced in Rufiji tilapia habitats (Shechonge et al., 2019). Recently, a genetic diversity study based on microsatellites provided evidence of extensive hybridization between the native Wami tilapia (*Oreochromis urolepis hornorum*; as mentioned earlier, recent evidence suggests to be the same species with Rufiji tilapia) and the introduced tilapia species raising concerns regarding the impact of introgression into the native populations (Shechonge et al., 2018).

Apart from being a species of high ecological value for Tanzanian aquatic habitats, Rufiji tilapia is economically important for both local fisheries and aquaculture activities. Rufiji tilapia is an attractive species for farming due to its high growth capacity, its inherent high salinity tolerance that could assist toward the expansion of the coastal aquaculture production in the country (Kajungiro, Mapenzi, et al., 2019), and the production of all‐male hybrids when crossed with female Nile tilapia (Mapenzi & Mmochi, 2016). Therefore, promoting Rufiji tilapia farming could result in the reduction of introduced non‐native tilapia species for aquaculture purposes mitigating biodiversity related concerns.

Reduced‐representation genotyping approaches constitute a powerful tool for conducting in‐depth population genetics studies for any species of interest. Following the introduction of restriction‐site‐associated DNA sequencing (Baird et al., 2008), a wide range of related methodologies utilizing restriction enzymes have been introduced like genotyping by sequencing (Elshire et al., 2011), ddRAD‐seq (Peterson, Weber, Kay, Fisher, & Hoekstra, 2012), 2b‐RAD (Wang, Meyer, McKay, & Matz, 2012), ezRAD (Toonen et al., 2013), quaddRAD (Franchini, Monné Parera, Kautt, & Meyer, 2017), and 2RAD/3RAD (Bayona‐Vásquez et al., 2019). The aforementioned platforms have been used extensively in studies on aquatic organisms focusing both in population genetic aspects (Andrews, Good, Miller, Luikart, & Hohenlohe, 2016) and in studying traits of interest for farming purposes (Houston et al., 2020; You, Shan, & Shi, 2020). ddRAD‐seq is one of the most commonly utilized member of the reduced‐representation family combining simplicity and cost efficiency during library construction (Peterson et al., 2012). Over the last years, ddRAD‐seq has been successfully utilized in a plethora of tilapia focussed studies investigating the underlying genetic structure of traits of economic value (Jiang et al., 2019; Li, Zhu, Gu, Lin, & Xia, 2019; Li et al., 2017; Palaiokostas et al., 2015; Taslima et al., 2020), for species‐specific SNPs (Syaifudin et al., 2019) and for deciphering the genetic diversity–population structure of wild and farmed populations (Kajungiro, Palaiokostas, et al., 2019; Moses et al., 2019).

The objective of the current study was to assess the genetic variation among 10 Rufiji tilapia populations of both wild (eight populations) and farmed (two populations) origin using ddRAD‐seq. Single nucleotide polymorphisms (SNPs) were identified across 195 animals and were subsequently used to estimate standard genetic diversity metrics both within and among populations, investigate for the existence of putative genetic clusters, test for the existence of isolation by distance, and assess the efficiency of predicting population of origin‐based only on the genomic profile using cross‐validation schemes. The aforementioned will facilitate both the conservation management of wild Rufiji tilapia populations and future breeding plans for aquaculture purposes where a broad genetic diversity is required for forming a base population.

## 2 MATERIALS AND METHODS

### 2.1 Sample collection and processing

**FIGURE 1:**
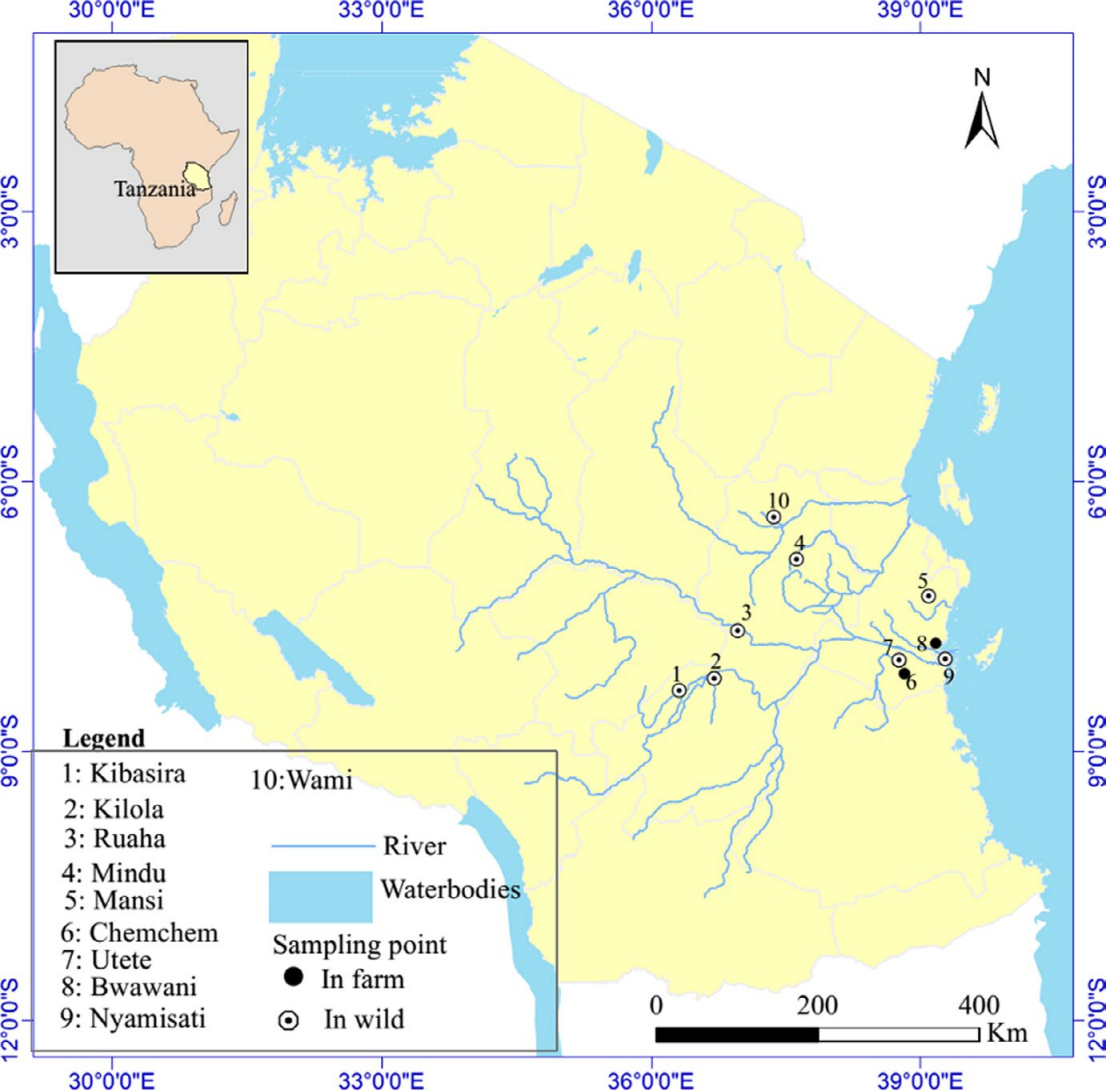
Sampling locations in Tanzania

Fish used in this study were collected from both wild and farmed environments in Tanzania mainland (Table 1). Sampling was performed using fishing nets (30 mm) with captured fish from 30 g and above being selected and conditioned for 24 hr at the sampling site or a nearby area before transportation. The sampled locations were selected based on prior available information regarding the *O. urolepis urolepis *distribution in Tanzania. In total, 10 different geographic locations were selected namely Nyamisati, Bwawani, Utete, Chemchem, Mansi, Mindu, Wami, Ruaha, Kibasira, and Kilola (Figure 1). The samples from Bwawani and Chemchem populations originated from fish farms located along the Rufiji River. In the case of the farmed population from Chemchem, available records suggest that the animals were in captivity for three consecutive generations, while in the case of Bwawani the sampled fish originated from the first generation in captivity. Species identification was performed using morphological criteria (Trewavas, 1983). In particular, coloration, size of jaws, and head shape were used to identify the Rufiji tilapia. More specifically, females and immature males had a light gray head, dark‐brown body with dark patches along the lateral line. On the other hand, mature males had a gray head, reddish‐pink fin margins and brownish‐golden upper parts. Besides, mature males had enlarged jaws and a concave‐shaped head. Regarding the population from the Wami river where *O.u. hornorum* is also endemic, identification of Rufiji tilapia was conducted based on skin pigmentation. In particular, males of *O.u.urolepis* are dark olive gray with pinkish upper lips and red fin margins, while *O.u. hornorum* males are entirely black with pale or black lips. In addition, *O.u. urolepis* females are silvery gray with a narrow pink edge on the dorsal fin, while the respective *O.u. hornorum* females have no pink edges. A total of 195 fish samples were collected and transported to the Institute of Marine Sciences Mariculture Centre (IMS‐MC) in Pangani, Tanzania.

**TABLE 1:** Origin of Rufiji tilapia (*Oreochromis urolepis urolepis*) populations

Population	Origin	*N*	Latitude	Longitude
Mindu	Wild	20	−7.434444	38.01722
Wami	Wild	20	−6.652222	37.60139
Bwawani	Farmed	20	−8.3175	39.46667
Kibasira	Wild	20	−8.535556	36.51694
Chemchem	Farmed	20	−8.651389	39.26889
Kilola	Wild	15	−8.318056	37.1675
Mansi	Wild	20	−7.4525	39.13528
Nyamisati	Wild	20	−8.301944	39.45056
Ruaha	Wild	20	−8.085556	37.60139
Utete	Wild	20	−8.633889	39.26889

### 2.2 DNA extraction and quantification

Fin clips of about 0.05 g were collected and preserved in 95% ethanol and stored at −20°C. Genomic DNA was extracted using QIAsymphony DSP DNA Mini Kit (Qiagen) and eluted into 100 μl of AE (EDTA) buffer (Qiagen) according to the manufacturer's tissue protocol and procedures. Quantification of DNA samples was done using a Qubit fluorimeter (Thermos Fisher Scientific, USA). Samples were diluted with TE buffer to 20 ng/μL followed by gel electrophoresis (1% agarose gel) to assess DNA quality.

### 2.3 ddRAD library preparation and sequencing

Two ddRAD libraries comprised of 96 and 99 individuals, respectively, were prepared according to Peterson et al. (2012), with minor modifications described in Palaiokostas et al. (2015). Briefly, each sample (20 ng/μl DNA) was digested at 37°C for 60 min with SbfI (recognizing the CCTGCA|GG motif) and SphI (recognizing the GCATG|C motif) high fidelity restriction enzymes (New England Biolabs; NEB), using 6 U of each enzyme per microgram of genomic DNA in 1 × Reaction Buffer 4 (NEB). Reactions (6 μl final volume) were then heat inactivated at 65°C for 20 min. Individual‐specific combinations of P1 and P2 adapters, each with a unique 5 or 7 bp barcode, were ligated to the digested DNA at 22°C for 120 min by adding 1 μl SbfI compatible P1 adapter (25 nM), 0.7 μl SphI compatible P2 adapter (100 nM), 0.06 μl 100 mmol/L rATP (Promega, UK), 0.95 μl 1 × Reaction Buffer 2 (NEB), 0.05 μl T4 ligase (NEB, 2 × 106 U/mL) with reaction volumes made up to 12 μl with nuclease‐free water for each sample. Following heat inactivation at 65°C for 20 min, the ligation reactions were slowly cooled to room temperature (over 1 hr), combined in a single pool (for one sequencing lane) and purified. Size selection (300–600 bp) was performed by agarose gel separation followed by gel purification and PCR amplification. A total of 100 μl of the amplified libraries (13–14 PCR cycles) were purified using an equal volume of AMPure beads. The libraries were eluted into 20 μl EB buffer (MinElute Gel Purification Kit, Qiagen). The libraries were sequenced at Edinburgh Genomics Facility, University of Edinburgh on an Illumina HiSeq 4000 instrument.

### 2.4 Sequence data analysis and SNP genotyping

Reads of low quality (*Q* < 20) and missing the expected restriction sites were discarded. The retained reads were aligned to the Nile tilapia reference genome assembly [GenBank accession number GCA_001858045.1 (Conte, Gammerdinger, Bartie, Penman, & Kocher, 2017)] using bowtie2 (Langmead & Salzberg, 2012). Stacks v2.5 (Rochette, Rivera‐Colón, & Catchen, 2019) was used to identify and extract single nucleotide polymorphisms (SNPs) using *gstacks* (settings: var‐alpha 0.001; gt‐alpha 0.001; min‐mapq 40). In the case where a single ddRAD locus had multiple SNPs, only the first encountered SNP was used for downstream analysis (‐‐write‐single‐snp). SNPs with minor allele frequency (MAF) < 0.05 and maximum heterozygosity > 0.7 across the tested samples were discarded. Moreover, the genotypes obtained for each individual were interrogated for the number of reads supporting each allele. Genotypes supported by fewer than 20 reads or where the coverage of one of the alleles was more than three times higher than the other allele were substituted as missing. Finally, only SNPs found in at least 75% of the samples in each population were retained for downstream analysis.

### 2.5 Genetic diversity within and among populations

General genetic variation metrics like mean observed (*H*_o_) and expected (*H*_e_) heterozygosity, nucleotide diversity (*π*), average individual inbreeding coefficients (*F*_is_), and the corresponding standard errors (*SE*) were estimated using the Stacks software v2.5 (Rochette et al., 2019). Pairwise *F*_st_ values among all tested populations and their confidence intervals (using 1,000 bootstraps) were estimated using the R package StAMPP (Pembleton, Cogan, & Forster, 2013).

### 2.6 Isolation‐by‐distance (IBD) analysis

The R package adegenet v2.1.1 (Jombart, Devillard, & Balloux, 2010) was used to evaluate the presence and magnitude of putative isolation by distance across the studied populations of wild origin (Table 1). The magnitude of the computed correlation between the estimated genetic distances (Edwards, 1971) among populations and their respective geographic locations (Euclidean distance) was assessed using the *mantel.randtest* function. Statistical significance was inferred through comparing the estimated correlations of the distance matrices through 100,000 random permutations under a scenario where spatial structuring is absent.

### 2.7 Genetic clusters and ancestry

Principal component analysis (PCA) was conducted using the R package adegenet v2.1.1 for visualization purposes and for gaining insights regarding the existence of genetic clusters. The existence of putative genetic clusters was further investigated using the discriminant analysis of principal components (DAPC) (Jombart et al., 2010) with the same R package. More specifically, PCA was initially applied, followed by a cross‐validation step using the *xvalDapc* function to select the optimal number of principal components (PCs). Thereafter, a discriminant analysis step was conducted using predetermined clusters from the PCs. The selection of the optimal number of clusters (*K*) was based on the elbow method (Jombart et al., 2010) in regard to the Bayesian information criterion (BIC) values for each tested value of *K*. Moreover, putative population admixture was investigated with Structure v.2.3.4 (Hubisz, Falush, Stephens, & Pritchard, 2009; Pritchard, Stephens, & Donnelly, 2000) using *K* values ranging from 2 to 5. Markov chain Monte Carlo (MCMC) of 100,000 iterations with a burn‐in period of 10,000 was carried out for each *K* value. For each tested *K* value, three independent MCMC samplings were performed. Evidence for the optimal number of clusters was based on the obtained posterior probability values (Pritchard et al., 2000). In addition, for deciding regarding the optimal number of genetic clusters, we used the Structure Harvester (Earl & vonHoldt, 2012) and CLUMPAK (Kopelman, Mayzel, Jakobsson, Rosenberg, & Mayrose, 2015) software.

### 2.8 Prediction of population origin based on the genomic profile

Cross‐validation schemes (fourfold) were performed using the R package adegenet v2.1.1 (Jombart et al., 2010) in order to test the utility of the SNP dataset for discriminating between (a) fish of farmed or wild origin and (b) fish originating from different geographic locations. Specifically, in the first cross‐validation scheme, the origin of 25% animals from wild and farmed origin was masked and treated as a test set, while the rest of the dataset was used for model training purposes. Predictions regarding the population of origin on the aforementioned test set were performed using information obtained through DAPC (*predict.dapc*) on the remaining training data set. The same procedure was followed for the second cross‐validation scheme where the origin of 25% of animals from each geographic population was masked and used as a test set. The entire procedure for both cross‐validation schemes was repeated ten times in order to minimize potential bias due to the stochasticity of sample allocation in the training/test datasets.

## 3 RESULTS

### 3.1 SNP identification using ddRAD sequencing

More than 320 million paired‐end reads were obtained. The sequenced reads were aligned to the Nile tilapia reference genome (GenBank accession GCA_001858045.2; Conte et al., 2017). Between 94% and 97% of the reads across the tested animals were aligned to the reference genome with approximately 16 million paired‐end reads being removed as unmapped. Additionally, approximately 71 million paired‐end reads were removed due to insufficient mapping quality (Phred‐scale mapping quality < 40). In total, 28,712 putative ddRAD loci were identified, out of which 4,719 contained one or more SNPs. The mean sequence coverage of the identified loci was approximately 105× (*SD*, 44×). Overall, 2,182 SNPs passed all quality control steps and were retained for downstream analysis. Finally, all 195 samples had fewer than 25% missing genotypes and were utilized for the subsequent analysis.

### 3.2 Genetic diversity within and among populations—isolation by distance

The expected heterozygosity (*H*_e_) and nucleotide diversity (*π*) estimates were largely indistinguishable with values for both parameters ranging from 0.10 to 0.37 (Table 2). Highest values were observed in the samples from Mindu (*H*_e_ = 0.22; *π* = 0.23) and Wami populations (*H*_e_ = 0.36; *π* = 0.37). On the other hand, the lowest values were observed in samples from Bwawani and Kibasira (*H*_e_ = 0.10; *π* = 0.10). Observed heterozygosity (*H*_o_) estimates ranged between 0.10 and 0.21 with the lowest values observed in samples from Bwawani and Kibasira and highest in samples from Mindu population. Moreover, regarding the inbreeding coefficient (*F*_is_), positive estimates were obtained for nine of the tested populations. After taking into account the corresponding standard error (*SE*) two populations showed suggestive evidence of putative loss of heterozygosity. The most striking difference was obtained in the Wami population (*F*_is_ = 0.42). An opposite trend was observed for the Mansi population (*F*_is_ = −0.03), suggesting a slight excess of heterozygotes. However, the corresponding *SE* was the highest among all tested populations (*SE* = 0.05).

**TABLE 2:** Estimates of genetic diversity

Population	*H*_e_ (*SE*)	*π* (*SE*)	*H*_o_ (*SE*)	*F*_is_ (*SE*)
Mindu	0.22 ± 0.003	0.23 ± 0.003	0.21 ± 0.003	0.04 ± 0.04
Wami	0.36 ± 0.004	0.37 ± 0.004	0.18 ± 0.003	0.42 ± 0.03
Bwawani	0.10 ± 0.003	0.10 ± 0.004	0.10 ± 0.004	0.01 ± 0.03
Kibasira	0.10 ± 0.003	0.10 ± 0.004	0.10 ± 0.004	0.01 ± 0.04
Chemchem	0.11 ± 0.003	0.11 ± 0.004	0.11 ± 0.004	0.01 ± 0.02
Kilola	0.11 ± 0.003	0.11 ± 0.004	0.10 ± 0.004	0.05 ± 0.01
Mansi	0.18 ± 0.003	0.18 ± 0.003	0.19 ± 0.003	−0.03 ± 0.05
Nyamisati	0.11 ± 0.004	0.11 ± 0.004	0.11 ± 0.004	0.02 ± 0.02
Ruaha	0.11 ± 0.003	0.11 ± 0.004	0.11 ± 0.004	0.02 ± 0.02
Utete	0.15 ± 0.003	0.15 ± 0.004	0.15 ± 0.004	0.01 ± 0.02

Note

*H*_e_ refers to expected heterozygosity; *H*_o_ refers to observed heterozygosity; *π* refers to nucleotide diversity; and *F*_is_ refers to inbreeding coefficient.

**FIGURE 2:**
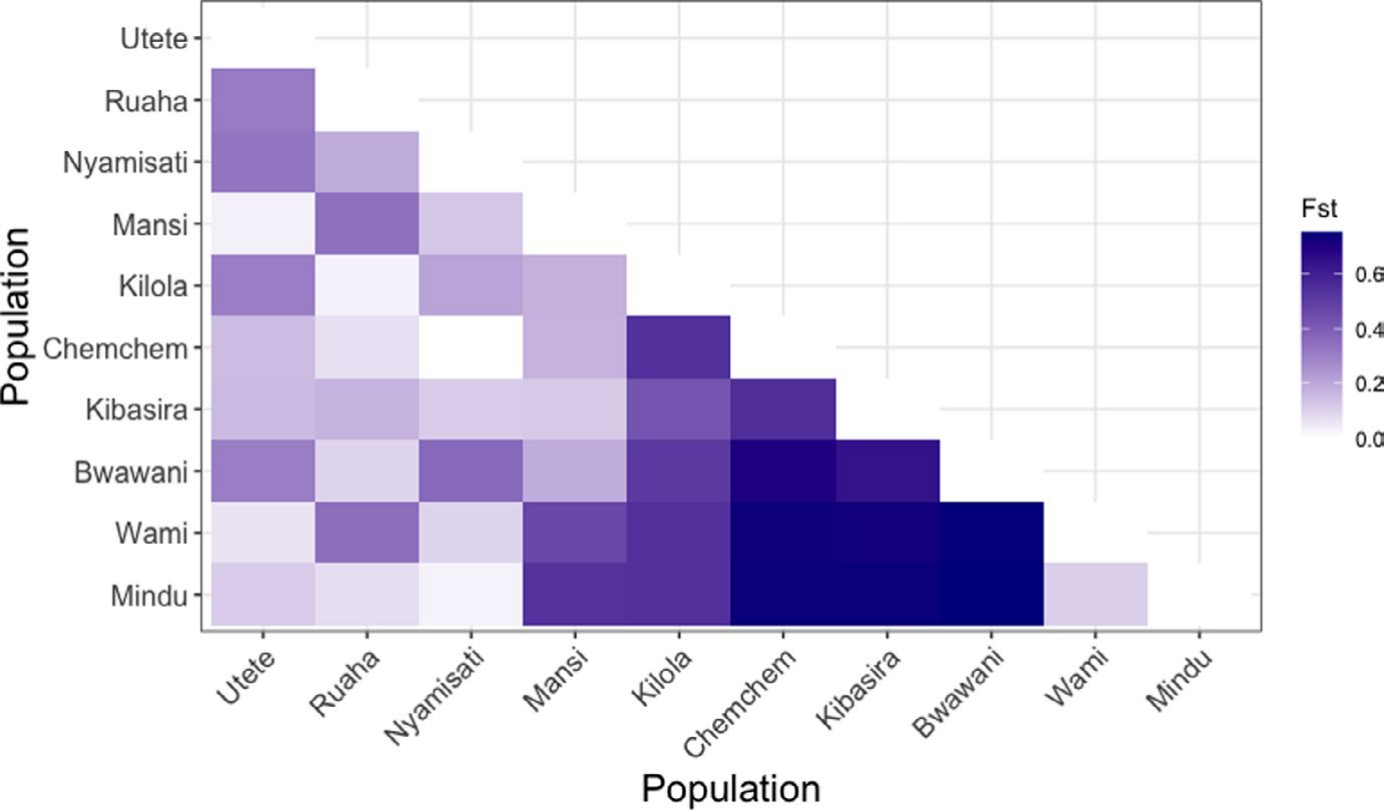
Genetic diversity among populations based on estimated *F*_st_ values. The Bwawani and Chemchem populations originated from fish farms located along the Rufiji River

The estimated genetic distances according to the *F*_st_ metric varied widely between 0.001 and 0.75 among the tested populations (Figure 2; Table S1). The highest genetic distance was observed between Mindu and the populations from Bwawani and Kibasira (*F*_st_ = 0.75). On the other hand, the lowest genetic distance was observed between the Kibasira and Kilola populations (*F*_st_ = 0.001).

**FIGURE 3:**
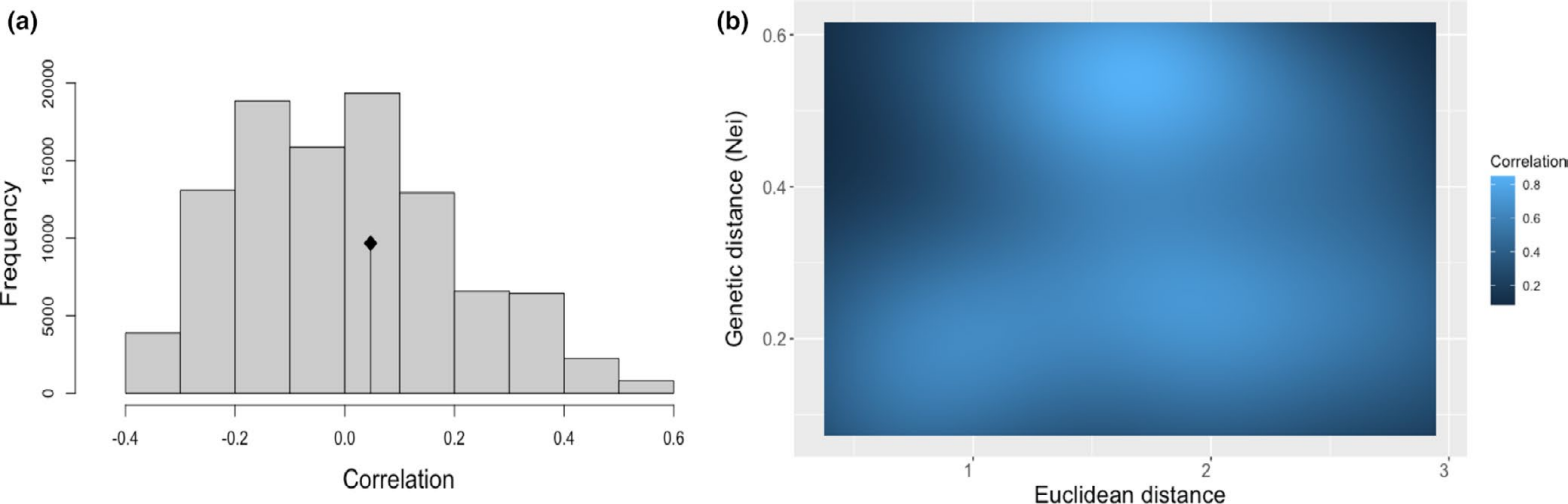
Isolation‐by‐distance analysis. (a) The original correlation among the distance matrices is represented by the dot. The histogram depicts the permuted correlation values under the absence of spatial structure. (b) Heatmap depicting the estimated correlation between the genetic and the Euclidean distance

The conducted isolation‐by‐distance analysis did not detect a statistically significant spatial pattern between the estimated genetic distances and the corresponding geographic locations on the studied wild populations. The correlation among the above was 0.05 with the corresponding p‐value after 100,000 permutations being 0.39 (Figure 3).

### 3.3 Population structure—admixture

**FIGURE 4:**
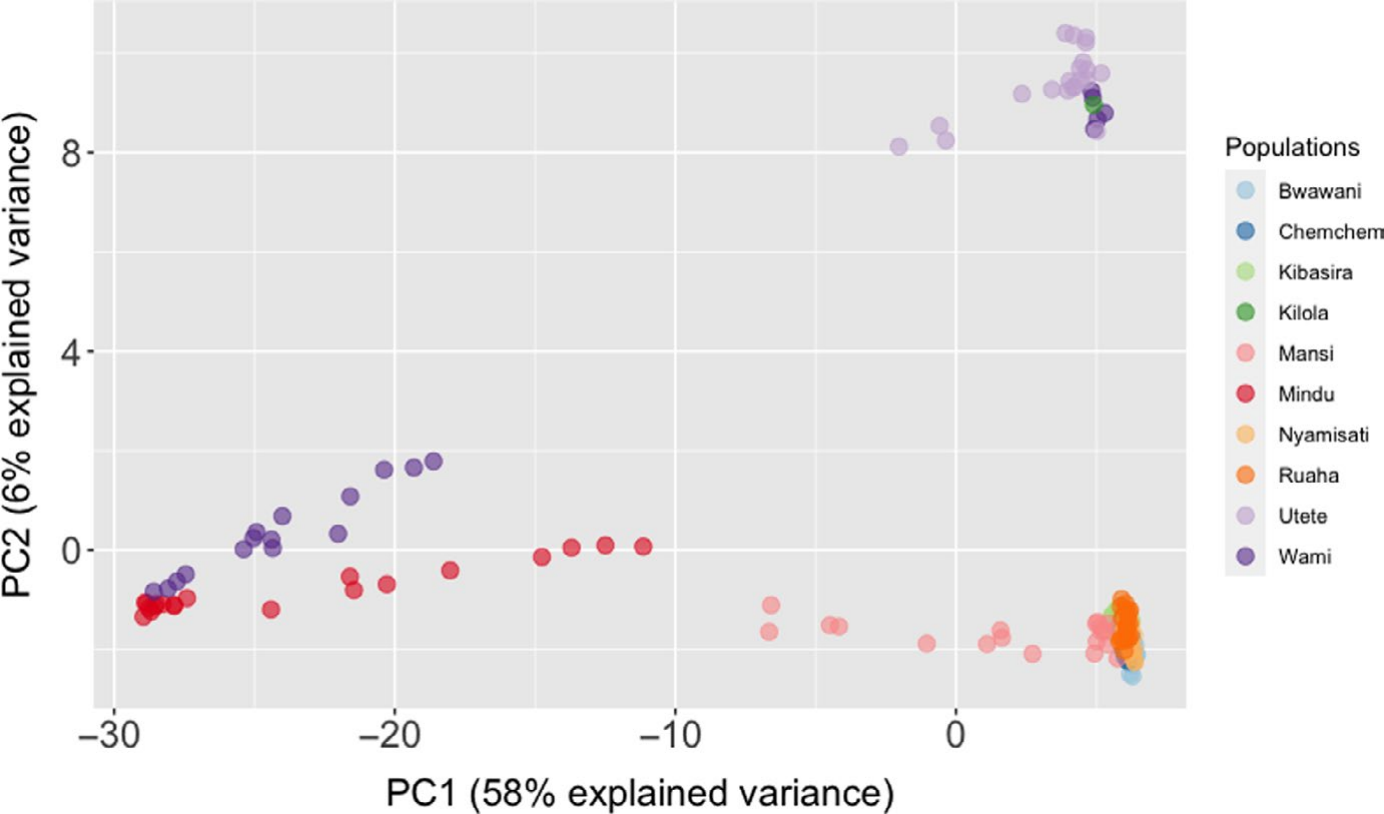
Principal component analysis (PCA) of Rufiji tilapia populations

**FIGURE 5:**
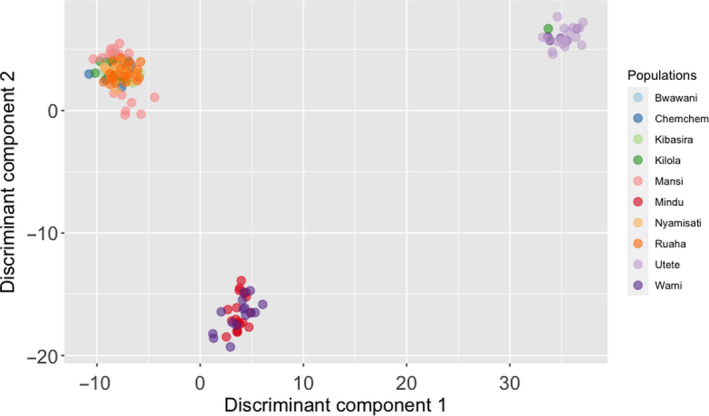
Discriminant analysis of principal components (DAPC) for Rufiji tilapia populations

Individual relationships within and between populations were visualized using PCA. The first and second principal components accounted for 58% and 6% of the observed variation, respectively. Overall, PCA indicated the existence of 3 groups among the sampled populations (Figure 4). Cross‐validation suggested that the optimal number of principal components for clustering was 40. Thereafter, DAPC further deciphered the putative genetic structure suggesting *K* = 3 to be the most probable number of genetic clusters (Figure 5; Figure S1). The first genetic cluster included Mindu and Wami populations, while the second cluster was comprised of the Kibasira, Kilola, Mansi, Bwawani, Ruaha, Nyamisati, and Chemchem. Finally, the last suggested cluster included the Utete population, one individual from Kilola and five individuals from Wami populations.

**FIGURE 6:**
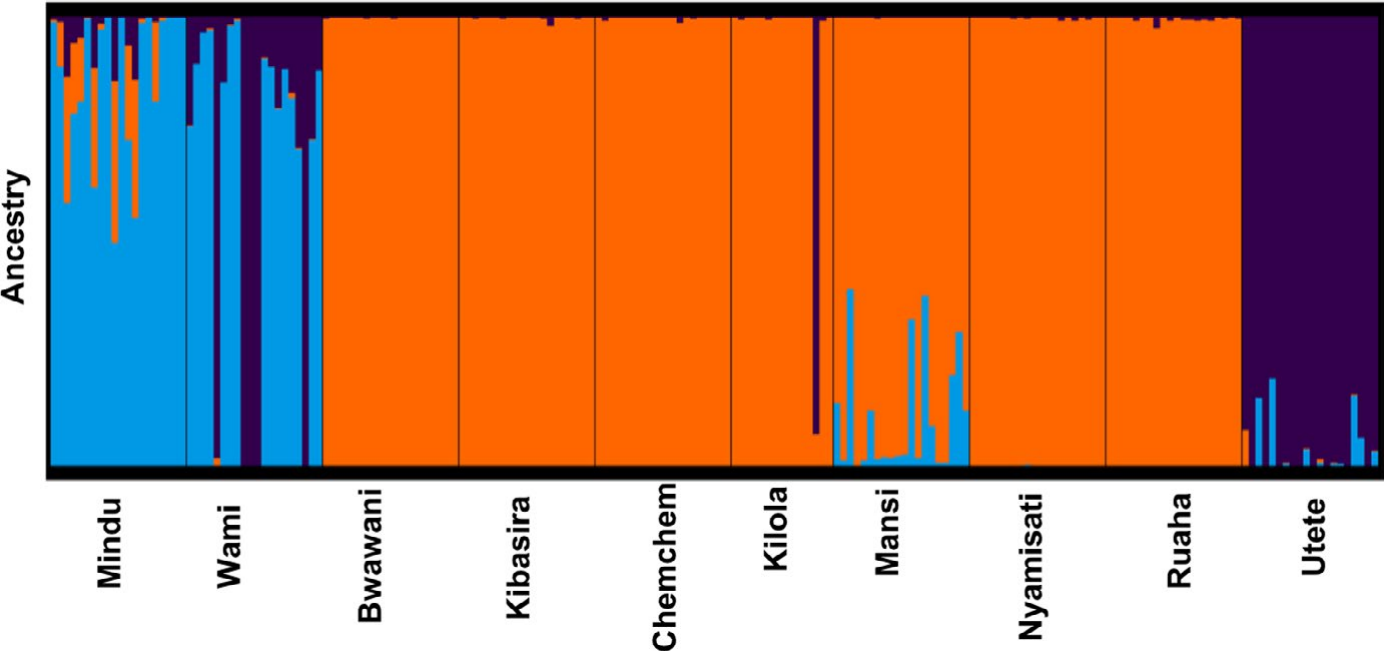
Ancestry analysis assigned individuals in clusters (*K* = 3). Each single vertical bar represents an individual and each color represents the probability that the individual is assigned to the respective gene pool. The Bwawani and Chemchem populations originated from fish farms located along the Rufiji River

Ancestry analysis provided further evidence regarding the existence of genetic clusters and potential admixture among the tested populations also indicating that *K* = 3 is the most probable number of clusters (Figure 6). Indication for admixture was observed between the Wami and Utete populations. Furthermore, admixture was suggested for the Mindu population and the genetic cluster comprised of Kibasira, Kilola, Mansi, Bwawani, Ruaha, Nyamisati, and Chemchem.

### 3.4 Origin prediction using the genomic profile

**FIGURE 7:**
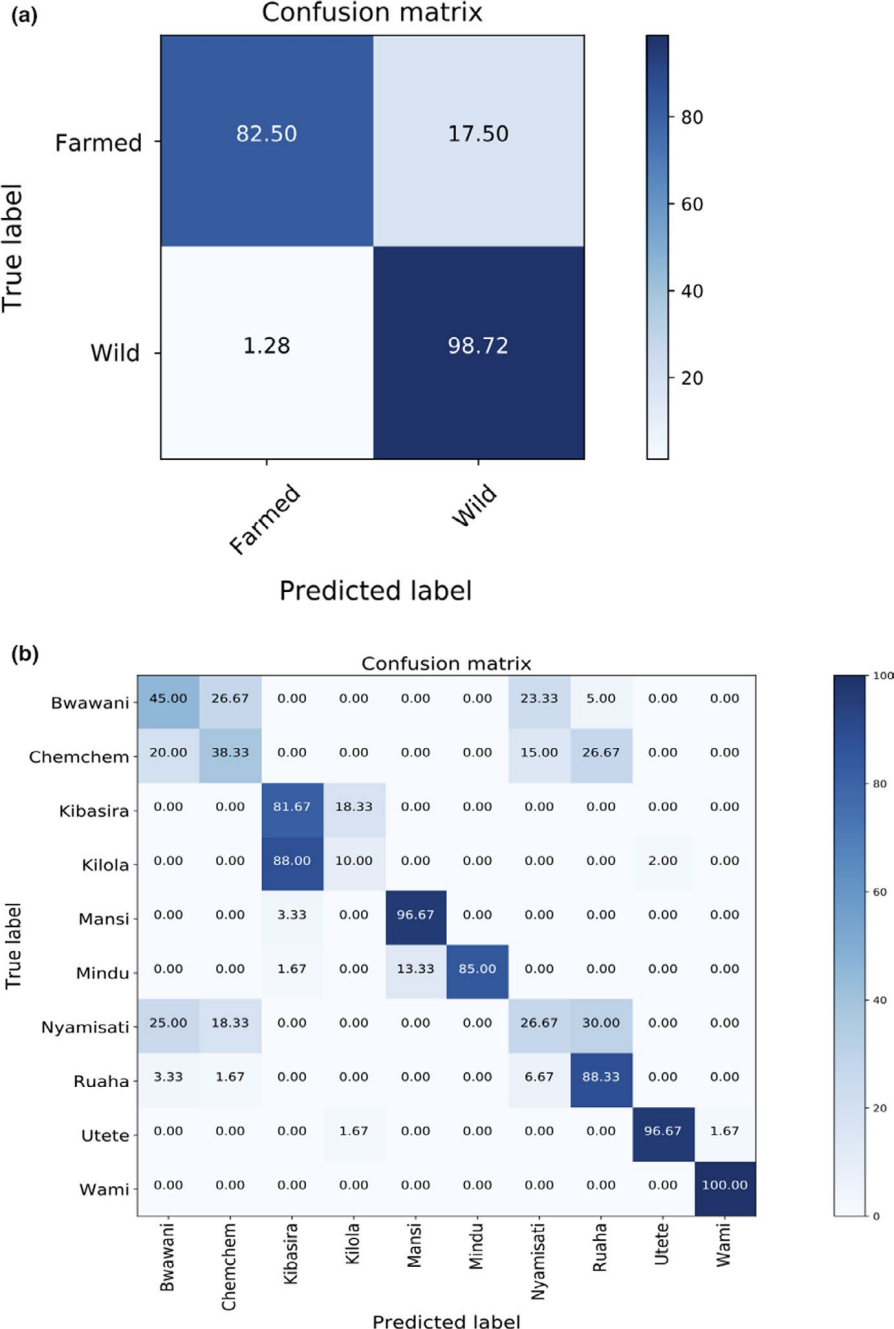
Confusion matrix for prediction efficiency (% of successful classification) of the SNP dataset using cross‐validation. (a) Fourfold cross‐validation to discriminate between farmed and wild origin. The origin of 25% randomly selected animals of wild and farmed origin was masked and used as a test set. Each population was considered of unknown origin. (b) Fourfold cross‐validation to predict population of origin. The origin of 25% randomly selected animals from each population was masked and used as a test set. The entire procedure in both (a) and (b) was repeated 10 times in order to minimize potential bias due to the initial sample allocation in the training/ test dataset. The diagonal contains the mean % percentage of correct population assignments for the overall cross‐validation scheme. Off‐diagonals contain the mean % percentage of wrong population allocations for each particular case

The utility of the SNP dataset to predict farmed versus wild and population of origin was tested using DAPC. In the fourfold cross‐validation, the mean successful assignment rate regarding farmed or wild origin on the test dataset was approximately 95% (Figure 7a). Regarding predictions for the population of origin, the overall successful classification was approximately 68% (Figure 7b). Classification success varied widely among populations with 100% for the Wami and only 10% for the Kilola population (Figure 7b).

## 4 DISCUSSION

In the current study, we obtained an in‐depth insight regarding the genetic variation within and among Rufiji tilapia populations in Tanzania using ddRAD‐seq. It is worth mentioning that in both the Mindu and the Ruaha reservoirs, the Rufiji tilapia is the only indigenous *Oreochromis* species (Eccles, 1992). While IUCN Red List of Threatened Species assessments exist for populations of several Oreochromis species in Tanzania, limited information is available regarding the status of Rufiji tilapia populations (Shechonge et al., 2019). Information regarding the genetic diversity and structure of either farmed or wild populations can assist toward their most suitable management and increase the efficiency of conservation activities. Reduced‐representation sequencing platforms like ddRAD‐seq are powerful tools for the aforementioned and have been widely applied in population genetic studies (McKinney, Larson, Seeb, & Seeb, 2017). The RAD‐seq family allows for high‐resolution studies of genetic diversity and relatedness at both population and individual levels (Lemopoulos et al., 2019; Palaiokostas et al., 2020). Moreover, reduced‐representation sequencing platforms do not suffer from ascertainment bias opposed to other genotyping platforms where a priori identified genetic markers are utilized.

It has to be pointed out that the identified SNPs used in our study were detected after aligning the sequenced reads in the Nile tilapia reference genome (GenBank accession GCA_001858045.2) which could entail a certain level of bias during SNP detection. However, the fact that more than 94% of the sequenced reads were aligned to the reference genome indicates that the subsequent SNP detection is robust. Furthermore, even though our approach would not have been able to identify Rufiji tilapia specific loci, the high percentage of aligned reads indicates that the latter would have been most likely a very small percentage with limited effect on the downstream analysis. It would worth also to stress the fact that Nile and Rufiji tilapias can produce fully fertile hybrids (Ulotu et al., 2016) when crossed together therefore indicating the similarity among the two species.

### 4.1 Genetic diversity within and among populations

According to the estimated genetic diversity metrics, the studied populations varied widely both in terms of *H*_e_ (0.10–0.36), *π* (0.10–0.37) and *H*_o_ (0.10–0.21). Compared to previous population genetics studies on aquatic organisms using RAD‐family genotyping protocols (Drinan et al., 2018; Lemopoulos et al., 2019; Sherman et al., 2020; Vendramin et al., 2016), the obtained genetic diversity metrics for several of the populations in our study lie on the lower range of the reported values (Table 2). Nevertheless, in comparison to our previous studies on farmed Nile tilapia populations in Tanzania (Kajungiro, Palaiokostas, et al., 2019; Moses et al., 2019) where the same ddRAD library preparation protocol was used, the obtained genetic diversity metrics were in general higher in the current study. Additionally, it is worth to point out that low levels of heterozygosity were obtained for several tilapia populations in an extensive study across West Africa (Lind et al., 2019). Interestingly, the farmed populations of our study (Bwawani; Chemchem) ranked among the lowest in terms of heterozygosity values. However, four of the wild populations (Kibasira; Kilola; Nyamisati; and Ruaha) had indistinguishable genetic diversity estimates compared to the farmed ones suggesting that no clear inference could be drawn regarding a potential loss of genetic diversity due to farming practices. On the other hand, the inbreeding coefficient (*F*_is_) indicated a potential loss of heterozygosity only for the wild population from Wami. In general, high *F*_is_ values indicate the existence of nonrandom mating or population subdivision (Allendorf & Luikart, 2007). Interestingly, concerns regarding the conservation of the unique genetic pool of endemic tilapias in the Wami water basin due to the introduction of nonendemic species have been documented recently (Shechonge et al., 2018). As documented also on previous occasions, introduced *Oreochromis* species can have a detrimental impact on endemic fish fauna (Angienda et al., 2011; Ndiwa, Nyingi, & Agnese, 2014) which could be the case for the Wami population of Rufiji tilapia.

The SNP dataset provided indications regarding the genetic distance among the tested Rufiji tilapia populations. Populations sampled from neighboring locations were in general of low genetic distance (Figure 1; Figure 2) with most obvious the case of Kibasira and Kilola (*F*_st_ = 0.001). However, several exceptions were observed with the most striking exception being the one between Chemchem and Utete populations where a moderate‐to‐high genetic distance (*F*_st_ = 0.34) was estimated. In general, *F*_st_ values below 0.05 indicate minimal genetic differentiation, while values above 0.15 indicate the existence of substantial genetic differentiation (Wright, 1978). A followed up isolation‐by‐distance analysis conducted on the wild populations did not detect evidence for existing spatial structure patterns among the sampled populations. To the best of our knowledge, no prior study investigated for putative spatial structure patterns of *Oreochromis* species in Tanzania. However, prior studies reported the existence of significant spatial genetic structure among *Oreochromis* populations across Africa (Bezault et al., 2011; Lind et al., 2019). The suspected uncontrolled movement of tilapia stocks among different locations in Tanzania (Kajungiro, Mapenzi, et al., 2019) could be a possible explanation for the observed absence of any statistically significant spatial structure among the studied populations. Nevertheless, it would be of primary importance to further verify the putative lack of spatial genetic structure we observed in future studies with larger number of samples per population.

### 4.2 Genetic structure of the tested populations

Bayesian and multivariate approaches were used in the current study in order to decipher the genetic structure and putative admixture among the tested populations. Both approaches supported the hypothesis of three unique genetic clusters among the populations under study (Figures 4, 5). To the best of our knowledge, no prior study investigated the existence of genetic structure among Rufiji tilapia. Therefore, the above information could guide the management of the wild resources and inform breeding initiatives for aquaculture purposes. Regarding the latter, in order to maximize the genetic diversity for a founding breeding population (Gjedrem, Robinson, & Rye, 2012) obtaining broodfish originating from all three genetic clusters could be a valid strategy. More specifically, the majority of samples from seven tested populations including the farmed ones (Kibasira, Kilola, Mansi, Bwawani, Ruaha, Nyamisati, and Chemchem) formed a unique genetic cluster, while the Wami and Mindu populations formed a separate genetic cluster (substantially differentiated according to obtained *F*_st_ values). As previously mentioned, Rufiji tilapia is the only endemic *Oreochromis* species in the Mindu reservoir (Shechonge et al., 2018); therefore, appropriate conservation management appears as a necessity on the aforementioned genetic cluster. Interestingly, the Utete population formed an isolated cluster that included one animal from Kilola and five animals from Wami populations. Moreover, ancestry analysis indicated the existence of admixture among the above populations. Nevertheless, taking into account the relatively small number of animals genotyped per population (*n* = 15–20) the possibility of sample mislabeling cannot be excluded especially in the case of the single animal from Kilola population that appeared genetically distant from its putative population of origin.

### 4.3 Prediction of population origin using SNP derived information

Overall, our SNP dataset proved highly efficient in discriminating between farmed and wild populations with approximately 95% of “putative” unknown samples being classified correctly. Considerable evidence suggests that hatchery rearing in various fish species can negatively affect key phenotypic traits associated with adaptation in the wild (Fraser, 2008). It is likely that the above could be even more evident in tilapias due to their relatively small generation interval (6 months or less to be reproductive mature under optimal environmental conditions). Furthermore, considering the fact that Tanzania is a hot spot for wild cichlid populations, it is evident that introgression with farmed strains could jeopardize the local adaptivity of the wild populations (Shechonge et al., 2018). It is worth mentioning that a recent study detected introgression between introduced *Oreochromis* species in Tanzania oriented for aquaculture practices and the critically endangered *Oreochromis jipe* (Bradbeer et al., 2019). Nevertheless, we need to acknowledge the fact that only two farmed populations were used in our study which limits our ability to draw definite conclusions.

The efficiency of the SNP dataset dropped remarkably (68% successful classification) in the scenario of predicting for population of origin. The drop in the accuracy of successful classification appears to be in line with the obtained genetic distance of the respective populations. The above was more pronounced in the case of Kilola and Kibasira where the proportion of correctly classified fish dropped to only 10% indicating that the two populations were highly similar (also supported from their estimated genetic distance and population structure). Moreover, a similar pattern was observed in the case of the farmed populations (Bwawani and Chemchem) and the respective wild populations of most likely putative origin (Nyamisati and Ruaha) suggested by our data. Aiming to acquire deeper insights and confirm that the reduction of successful classification for predicting population of origin was due to the low genetic distance between some of the studied populations, we tested our dataset in a theoretical scenario aiming to predict for genetic cluster. In particular, since our analysis suggested the existence of three distinct genetic clusters, we followed the same cross‐validation scheme as before for forming training and validation sets on each putative genetic cluster (fourfold cross‐validation). The above allowed us to obtain close to 100% successful classification on the test dataset.

Moreover, a similar approach was followed in our prior studies on Nile tilapia strains (mainly of farmed origin) where the SNP information allowed for correctly classifying between 77% and 97% of the tested dataset to the respective population of origin (Kajungiro, Palaiokostas, et al., 2019; Moses et al., 2019). However, in the aforementioned studies we used mainly farmed populations of more pronounced genetic distance as opposed to the Rufiji populations of the current study which facilitated their discrimination in the followed cross‐validation schemes. Therefore in this particular instance, the SNP dataset was less efficient on predicting for population of origin largely due to the fact that some of the tested populations proved to be less divergent than the aforementioned Nile tilapia populations. Nevertheless, we need to acknowledge the fact that a low‐density genotyping approach was followed in our study which could limit our ability to discriminate between populations of low genetic distance. Therefore, high‐density genotyping approaches through the application of either more frequent cutting restriction enzymes or the recently developed open access tilapia SNP array (Peñaloza et al., 2020) could be of value for predicting with higher accuracy the population of origin even among closely related samples.

## 5 CONCLUSIONS

The current study is the first attempt of investigating the genetic diversity status of Rufiji tilapia populations using high‐throughput sequencing‐based platforms. Overall, the ddRAD‐seq derived SNP dataset was applied in a wide range of analysis deciphering the underlying genetic diversity and structure among the studied populations. The identified genetic structure would be of value both for conservation purposes and for future aquaculture breeding practices aiming to establish base populations with the highest amount of genetic diversity. Finally, taking into consideration the desirable traits of Rufiji tilapia for farming purposes studies of common garden experiments between Rufiji and introduced Nile tilapia would be valuable for informing future breeding plans targeting the productivity increase of Tanzanian aquaculture.

## CONFLICT OF INTEREST

The authors declare no conflict of interest.

## AUTHOR CONTRIBUTIONS

**Christer S. Nyinondi:** Formal analysis (equal); investigation (equal); methodology (equal); software (equal); writing – original draft (equal). **Matern S. P. Mtolera:** Conceptualization (equal); funding acquisition (equal); investigation (equal); methodology (equal); writing – review and editing (equal). **Aviti J. Mmochi:** Conceptualization (equal); methodology (equal); writing – review and editing (equal). **Fernando A. Lopes Pinto:** Investigation (equal); writing – review and editing (equal). **Ross Houston:** Conceptualization (equal); funding acquisition (equal); methodology (equal); supervision (equal); writing – review and editing (equal). **Dirk J. de Koning:** Conceptualization (equal); methodology (equal); project administration (equal); writing – review and editing (equal). **Christos Palaiokostas:** Conceptualization (equal); methodology (equal); supervision (equal); writing – review and editing (equal).

## ETHICAL APPROVAL

The current study was carried out in accordance with the law on the protection of animals against cruelty (Act No. 12/1974. of the United Republic of Tanzania) upon its approval by the Department of Zoology and Wildlife Conservation, University of Dar es salaam. All the permits required to sample wild animals in Tanzania adhered to the Research clearance from Tanzania Commission for Science and Technology (COSTECH).

## Supporting information

Figure S1

Table S1

## Data Availability

The aligned reads in the format of bam files were deposited in the National Centre for Biotechnology Information (NCBI) repository under project ID PRJNA518067. Supplementary information regarding all pairwise *F*_st_ values with their corresponding confidence intervals and the BIC values for each test number of clusters (*K*) were deposited in Dryad (https://doi.org/10.5061/dryad.x0k6djhgq).
