# Supplementary figures and images for: Assessing the genetic diversity of farmed and wild Rufiji tilapia (Oreochromis urolepis urolepis) populations using ddRAD sequencing
Source: Ecol Evol. 2020 Aug 18;10(18):10044–56. doi: 10.1002/ece3.6664 (PMC7520224; doi:10.1002/ece3.6664)

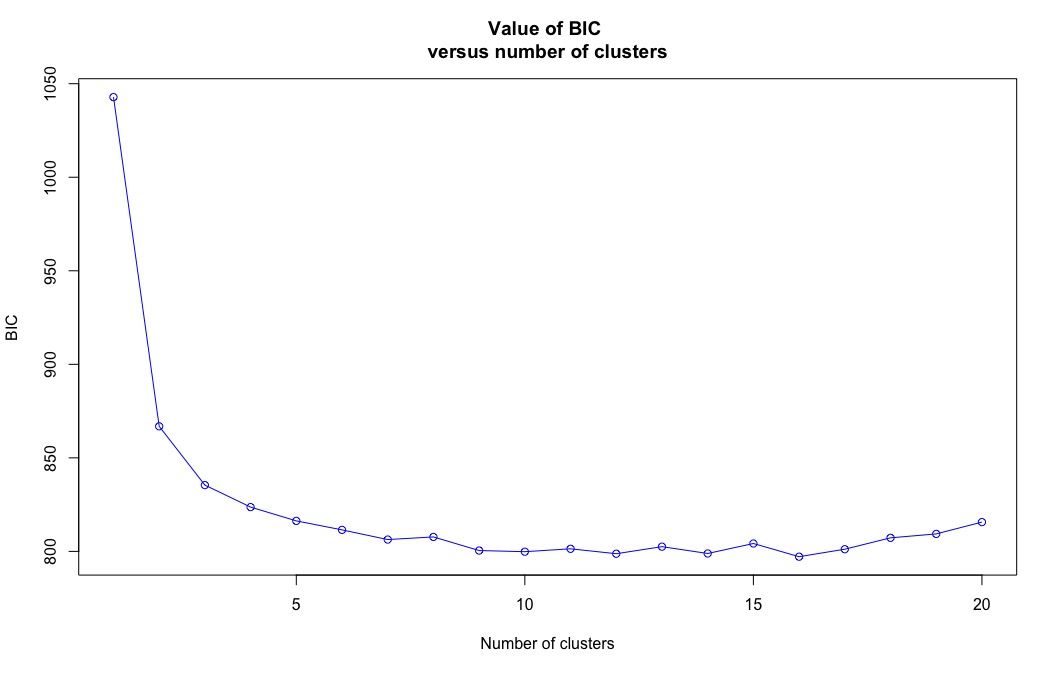

Supplement: Supplementary file 1 — Figure S1 [file ECE3-10-10044-s001.jpeg]
