# Supplementary material for: Assessing the genetic diversity of farmed and wild Rufiji tilapia (Oreochromis urolepis urolepis) populations using ddRAD sequencing
Source: Ecol Evol. 2020 Aug 18;10(18):10044–56. doi: 10.1002/ece3.6664 (PMC7520224; doi:10.1002/ece3.6664)
Supplement: Supplementary file 2 — Table S1 [file ECE3-10-10044-s002.docx]

**Supplementary**

**Table S1**. Pairwise genetic distances between Rufiji tilapia populations

| **Population 1** | **Population 2** | **F_st_** | **CI** |
| --- | --- | --- | --- |
| Mindu | Wami | 0.11 | 0.10 - 0.11 |
| Mindu | Bwawani | 0.75 | 0.74 - 0.76 |
| Mindu | Kibasira | 0.75 | 0.74 - 0.76 |
| Mindu | Chemchem | 0.74 | 0.73 - 0.75 |
| Mindu | Kilola | 0.73 | 0.72 - 0.74 |
| Mindu | Mansi | 0.64 | 0.64 - 0.65 |
| Mindu | Nyamisati | 0.74 | 0.73 - 0.75 |
| Mindu | Ruaha | 0.74 | 0.73 - 0.75 |
| Mindu | Utete | 0.70 | 0.69 - 0.71 |
| Wami | Bwawani | 0.55 | 0.54 - 0.56 |
| Wami | Kibasira | 0.54 | 0.53 - 0.55 |
| Wami | Chemchem | 0.54 | 0.53 - 0.55 |
| Wami | Kilola | 0.51 | 0.50 - 0.52 |
| Wami | Mansi | 0.43 | 0.42 - 0.43 |
| Wami | Nyamisati | 0.54 | 0.53 - 0.55 |
| Wami | Ruaha | 0.53 | 0.52 - 0.54 |
| Wami | Utete | 0.47 | 0.46 - 0.48 |
| Bwawani | Kibasira | 0.19 | 0.17 - 0.21 |
| Bwawani | Chemchem | 0.12 | 0.11 - 0.13 |
| Bwawani | Kilola | 0.17 | 0.15 - 0.19 |
| Bwawani | Mansi | 0.18 | 0.16 - 0.19 |
| Bwawani | Nyamisati | 0.03 | 0.02 - 0.03 |
| Bwawani | Ruaha | 0.10 | 0.08 - 0.11 |
| Bwawani | Utete | 0.37 | 0.34 - 0.39 |
| Kibasira | Chemchem | 0.11 | 0.10 - 0.13 |
| Kibasira | Kilola | 0.001 | -0.003- 0.004 |
| Kibasira | Mansi | 0.21 | 0.19 - 0.23 |
| Kibasira | Nyamisati | 0.13 | 0.11 - 0.14 |
| Kibasira | Ruaha | 0.08 | 0.06 - 0.09 |
| Kibasira | Utete | 0.35 | 0.33 - 0.37 |
| Chemchem | Kilola | 0.09 | 0.08 - 0.11 |
| Chemchem | Mansi | 0.17 | 0.16 - 0.19 |
| Chemchem | Nyamisati | 0.07 | 0.06 - 0.08 |
| Chemchem | Ruaha | 0.03 | 0.03 - 0.04 |
| Chemchem | Utete | 0.34 | 0.32 - 0.36 |
| Kilola | Mansi | 0.19 | 0.17 - 0.21 |
| Kilola | Nyamisati | 0.11 | 0.10 - 0.13 |
| Kilola | Ruaha | 0.06 | 0.05 - 0.07 |
| Kilola | Utete | 0.31 | 0.29 - 0.33 |
| Mansi | Nyamisati | 0.15 | 0.14 - 0.17 |
| Mansi | Ruaha | 0.15 | 0.14 - 0.16 |
| Mansi | Utete | 0.31 | 0.29 - 0.33 |
| Nyamisati | Ruaha | 0.03 | 0.03 - 0.04 |
| Nyamisati | Utete | 0.33 | 0.31 - 0.35 |
| Ruaha | Utete | 0.32 | 0.30 - 0.34 |

CI refers to confidence intervals (95%; 1000 bootstraps)
